# The Potential Renoprotective Effect of Sugammadex in Renal Ischemia-reperfusion Injury

**DOI:** 10.4274/TJAR.2025.251887

**Published:** 2025-12-22

**Authors:** Vildan Kölükçü, Mehtap Gürler Balta, Ahmet Tuğrul Şahin, Ali Genç, Velid Unsal, Fatih Fırat, Fikret Gevrek, Asiye Yancı, Ahmet Burak Gürpınar

**Affiliations:** 1 Tokat Gaziosmanpaşa University Faculty of Medicine, Department of Anaesthesiology and Reanimation, Tokat, Türkiye; 2 Mardin Artuklu University, Faculty of Health Sciences, and Central Research Laboratory, Mardin, Türkiye; 3 Tokat Gaziosmanpaşa University Faculty of Medicine, Department of Urology, Tokat, Türkiye; 4 Tokat Gaziosmanpaşa University Faculty of Medicine, Department of Histology and Embryology, Tokat, Türkiye; 5 Tokat Gaziosmanpaşa University Faculty of Medicine, Department of Biochemistry, Tokat, Türkiye

**Keywords:** Ischaemia-reperfusion injury, renal, rat, sugammadex

## Abstract

**Objective:**

We aimed to evaluate the effectiveness of sugammadex on renal tissue for against ischemia-reperfusion injury.

**Methods:**

Twenty-one Wistar albino strain female rats were divided into three groups. The first group functioned as the control cohort for comparison. In Groups 2 and 3, a renal ischemia-reperfusion model was established. Moreover, following the cessation of ischemia, the rats in Group 3 were intravenously administered sugammadex at a dose of 4 mg kg^-1^. Blood and tissue samples were subsequently collected for analysis.

**Results:**

Biochemical analyses revealed a notable increase in the enzymatic activities of glutathione peroxidase and superoxide dismutase in Group 3 relative to Group 2 (*P*< 0.001 and *P*=0.015, respectively). Additionally, the concentration of malondialdehyde was found to be significantly reduced in Group 3 relative to Group 2 (*P*=0.004). Group 3 exhibited a substantial decrease in tumor necrosis factor-alpha, interleukin 6, and interleukin 1 beta levels when compared to Group 2 (*P*=0.021, *P*=0.006, and *P*=0.016 respectively). Group 2 exhibited the highest concentrations of neutrophil gelatinase-associated lipocalin and kidney injury molecule-1 (*P*< 0.001 and *P*=0.015, respectively). Similarly, the histopathologic tissue damage was the most prominent in Group 2 (*P*< 0.001).

**Conclusion:**

Sugammadex plays a protective role against ischaemia-reperfusion injury in renal tissue.

**Main Points**

• A sudden deterioration in renal function, known as acute renal failure, results in the retention of metabolic waste and disruptions in fluid and electrolyte regulation.

• One of the primary contributors to acute renal failure is renal ischemia-reperfusion injury.

• Sugammadex is a pharmacological agent widely used in general anaesthesia practices to reverse the effects of muscle relaxants.

• Our study’s biochemical and histopathological data suggest that sugammadex provides substantial protection against renal ischemia-reperfusion injury.

## Introduction

Acute renal failure is a medical disorder marked by an abrupt decline in kidney function and is commonly encountered in emergencies and intensive care units.^1^ Approximately 5% of long-term hospitalized patients suffer from pathologies associated with acute renal failure.^2^ One of the primary contributors to acute renal failure is renal ischemia-reperfusion injury.¹ Renal ischemia is defined as a temporary reduction or interruption of renal blood flow. If renal ischemia persists for a long time, cellular integrity is disrupted, and cell death occurs due to excessive accumulation of toxic metabolites. Reperfusion is required to clear toxic metabolites and to maintain tissue viability. Paradoxically, detrimental changes occur much more severely during reperfusion than during ischemic injury. This condition is defined as ischemia-reperfusion injury. The kidneys are quite sensitive to ischemia-reperfusion injury: tubular damage, atrophy, and dilation in tubules are the most common histopathological findings.³ Several factors, including trauma, shock, infarction, sepsis, aortic dissection, and urologic surgery, contribute to the etiology of reperfusion injury.^1,4^ There is limited knowledge about the mechanism of renal ischemia-reperfusion injury. Multiple factors contributing to its pathogenesis, such as oxidative stress, epithelial cell dysfunction, inflammation, cellular necrosis, tubular obstruction, and apoptosis, have been identified by *in vivo* studies as well as by in *in vitro* studies in recent years.^5,6^ Furthermore, renal ischemia-reperfusion injury continues to be linked with mortality rates reaching as high as 79%.^7^ Renal ischemia-reperfusion is a topic that is intensively studied to minimize the effects on patients.^8^

Sugammadex is a pharmacological agent widely used in general anaesthesia practices to reverse the effects of muscle relaxants, such as rocuronium and vecuronium. This molecule, belonging to the gamma-cyclodextrin group and containing eight sugar rings, is widely used in operating rooms for purposes such as post-operative extubation and reversing newly developed blocks in patients who cannot be intubated or ventilated.^9,10^ In addition, recent experimental studies have documented that sugammadex mitigates the damage caused by ischemia-reperfusion injury by suppressing inflammation and enhancing antioxidant enzyme activity.^10,11^ The most important advantage of sugammadex is that it can provide fast and effective reversal in anaesthesia practice involving difficult airway ventilation.⁹ We thought that evaluating the positive effects of sugammadex, which provides significant advantages to anaesthesia, on tissue oxidative damage from a novel perspective would make important contributions to the literature.

The hypothesis of this study is that sugammedex may reduce renal ischemia reperfusion injury and have nephroprotective effects in a rat renal hypoxia model by showing anti-inflammatory and antioxidant activity. Although the renoprotective effect of sugammadex in renal ischemia-reperfusion injury has been previously evaluated histopathologically, this study aims, for the first time in the English literature, to investigate the potential protective role of sugammadex using both biochemical and histopathological analyses.

## Methods

### Experimental Animals and Groups

This research involved 21 female albino Wistar rats, each with a weight ranging from 220 to 400 g. The animals were kept in standard cages under strictly controlled conditions, with humidity levels maintained between 45% and 55%, while the ambient temperature was regulated at 20-22 °C. A 12-hour alternating light/dark cycle was applied throughout the study. Vital signs (heart rate, breathing rate, and depth) were stable and similar for all rats throughout the experiment. The experiment adhered to the National Institutes of Health guidelines for animal research and received approval from the Tokat Gaziosmanpaşa University Rectorate, Animal Experiments Local Ethics Committee (approval no.: 2024 HADYEK-13, date: 15.08.2024).

### Experimental Method

In the operating room, all experimental animals received an intraperitoneal injection of ketamine hydrochloride (50 mg kg^-1^) combined with xylazine (7.5 mg kg^-1^) to induce general anaesthesia. After the appropriate depth of anaesthesia was obtained, the abdominal hairs of all rats were shaved in the supine position, and sterile conditions were met by using povidone-iodine and sterile gauze. Subsequently, all experimental animals underwent laparotomy with a midline incision of 3 cm. The right nephrectomy was performed.^12^ Following nephrectomy, this procedure was conducted on all rats, and the experimental animals were allocated into three groups.

**Group 1:** This cohort functioned as the control cohort. The left renal pedicle was separated, and a left nephrectomy was performed without using any additional surgical intervention or pharmacological agent.

**Group 2:** This cohort functioned as the ischemia-reperfusion group. The left renal pedicle was separated and subjected to clamping for 45 minutes. A left nephrectomy was performed 6 hours after the clamp was taken out.^12^

**Group 3:** This cohort functioned as the treatment group. The same procedures applied in Group 2 were applied in Group 3. Additionally, 4 mg kg^-1^ of sugammadex was delivered intravenously to the rats in this group following the termination of ischemia.^10^

Fluid and heat balance was maintained very precisely throughout all interventions. Heparin (100 U kg^-1^) was administered intraperitoneally at the beginning of the procedures to prevent renal artery thrombosis.^13^ Upon completion of the experiment, blood samples were collected from all rats via the inferior vena cava, followed by a left nephrectomy. Finally, cervical dislocation was applied to all experimental animals to terminate their vital functions.

There were no human subjects in this study and informed consent is not applicable.

### Biochemical Analysis

### Preparation of Samples

Renal tissues and serum specimens were preserved at -80 °C. Half of the kidney tissue taken was separated for enzyme-linked immunosorbent assay (ELISA) testing and the other half for oxidative stress parameters. Only the supernatant was obtained for ELISA testing from kidney tissue. Kidney tissue specimens were homogenized on ice using a 50 mM Tris-HCl buffer solution (pH=7.4) at a dilution ratio of 1:10. Malondialdehyde (MDA) concentrations were quantified in the homogenized samples. A fraction of the homogenates underwent centrifugation at 3,200×g for about 15 minutes at 4 °C , yielding supernatant fractions. The enzymatic activities of superoxide dismutase (SOD) and glutathione peroxidase (GSH-Px) were assessed in the collected supernatants. Protein concentrations were evaluated in both homogenized samples and supernatant fractions.

### Measurement of GSH-Px and SOD Activity in Kidney Tissue

SOD enzyme activity was evaluated in accordance with the methodology outlined by Sun et al.,^14^ while GSH-Px levels were quantified following the protocol proposed by Paglia and Valentine. The enzymatic activities of both were quantified as units per gram of tissue protein (U g protein).

### Measurement of MDA Levels in Kidney Tissue

The quantification of MDA levels is based on its reaction with thiobarbituric acid at 90 °C, leading to the formation of a pink chromogen. This reaction product is subsequently measured using spectrophotometry at 532 nm. The obtained values are presented as (nmol g wet tissue) determined through a standard calibration curve generated from serial dilutions of 1,1,3,3-tetramethoxypropane.^16^

### Measurement of Protein in Tissues

Tissue protein levels were quantified with bovine serum albumin serving as the standard reference.^17^

Measurement of tumor necrosis factor-alpha (TNF-alpha), interleukin-6 (IL-6), interleukin-1beta (IL-1beta), kidney injury molecule-1 (KIM-1) and neutrophil gelatinase-associated lipocalin (NGAL).

Measurements were obtained by Thermo Scientific’s Multiskan FC photometric microplate reader. Kidney tissue was separated for KIM-1 and NGAL measurement. Kidney tissues were rinsed with ice-cold phosphate buffer solution (PBS) (0.01 N, pH=7.4) to remove excess blood. Kidney tissue specimens were homogenized on ice under cold conditions, using PBS at a rate of 1/10. Kidney injury molecule-1 and NGAL were investigated in supernatants obtained after homogenization. The results are expressed as ng protein.

The concentrations of TNF-alpha, IL-6 and IL-1beta in serum samples were measured through ELISA kits following the manufacturer’s guidelines. Results are expressed as ng l^-1^ and pg mL^-1^ respectively.

### Histopathological Analysis

Renal tissue specimens were maintained in a 4% buffered neutral formalin solution for 72 hours to facilitate histological examination. Following fixation, the kidneys were continuously rinsed with running water throughout the day, dehydrated through a sequential series of alcohol concentrations, and cleared using xylene baths. Subsequently, paraffin infiltration was performed in three separate paraffin baths at 60 °C. The tissues were then embedded in paraffin in the same orientation, and sectioned into blocks. Consecutive thin serial sections (5 μm thickness) of the paraffin-blocked kidneys were taken using a rotary microtome (Leica RM2135, Germany) and mounted on slides with ground edges for hematoxylin and eosin staining. These slides were then prepared for histopathological analysis.

Histological slides were examined blindly using a light microscope (Nikon Eclipse 200, Japan) at 40× magnification by a single experienced histopathologist. Renal tissue samples were assessed for overall structural integrity, parenchymal and stromal injury, tubular architecture, vascular congestion, and necrotic alterations. The severity of these pathological changes was graded semi-quantitatively on a scale from 1 to 4. Specifically, a score of 1 (none) denotes no detectable damage; a score of 2 (mild) represents minor alterations, including epithelial flattening, tubular dilation, nuclear dropout, and brush border loss; a score of 3 (moderate) signifies moderate injury characterized by focal coagulative necrosis; and a score of 4 (severe) corresponds to infarction or extensive tissue damage.^18^

### Statistical Analysis

Descriptive statistical analyses were performed to outline the general characteristics of the study groups. Continuous variables were represented as mean ± standard deviation along with median (minimum-maximum). Intergroup differences in variables were assessed using One-Way Analysis of Variance (ANOVA). Further comparisons were carried out using the post-hoc Tukey HSD test when the variances of the groups are equal (homogeneous variance) or Tamhane’s T2 test when the variances of the groups are not equal (heterogeneous variance). *P *values were deemed statistically significant when below 0.05. Statistical analyses used commercially available software (IBM SPSS Statistics 22, SPSS Inc., an IBM Company, Somers, NY).

## Results

**Figure 1:**
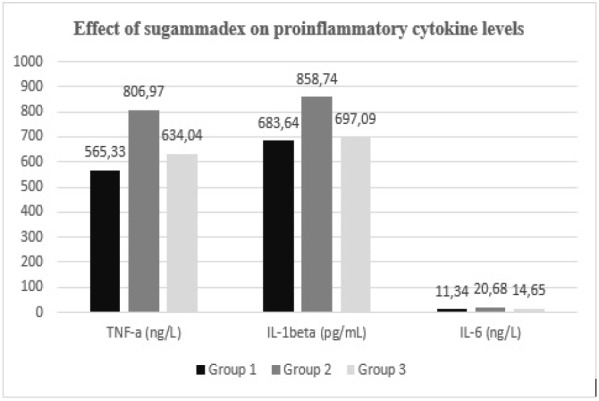
Comparative graphic representation of TNF-alpha, IL-1beta and IL-6 levels. TNF-alpha, tumor necrosis factor-alpha; IL, interleukin.

**Figure 2:**
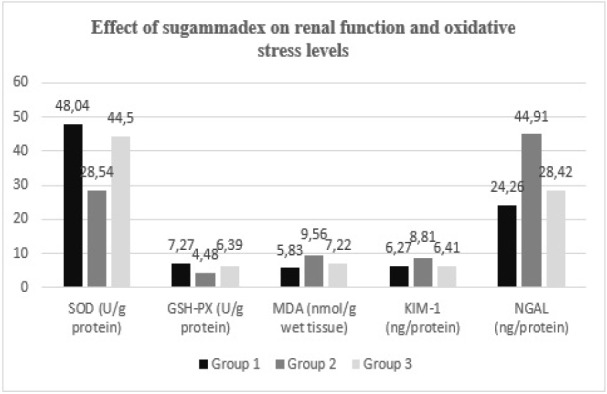
Comparative graphic representation of SOD, GSH-Px, MDA, KIM-1 and NGAL levels. SOD, superoxide dismutase; GSH-Px, glutathione peroxidase; MDA, malondialdehyde; KIM-1, kidney injury molecule-1; NGAL, neutrophil gelatinase-associated lipocalin.

Biochemical analysis of blood samples revealed a reduction in pro-inflammatory cytokine levels, including IL-1β, IL-6, and TNF-α, in Group 3 relative to Group 2 (*P*=0.016, *P*=0.006, and *P*=0.021, respectively) (Table 1, Figure 1). Tissue biochemical assessments demonstrated that Group 2 exhibited the lowest levels of GSH-Px and SOD activity (*P *< 0.001 and *P*=0.003, respectively). However, antioxidant enzyme levels were significantly elevated in Group 3 relative to Group 2 (*P* < 0.001 and *P*=0.015, respectively). The highest concentrations of NGAL and KIM-1 were observed in Group 2 (*P* < 0.001 and *P*=0.015, respectively), whereas a significant reduction in these markers was noted in Group 3 relative to Group 2 (*P* < 0.001 and *P*=0.034, respectively). The mean MDA level in Group 2 was measured at 9.56±1.81, and it significantly decreased in Group 3 (*P*=0.004) (Table 2, Figure 2).

**Figure 3:**
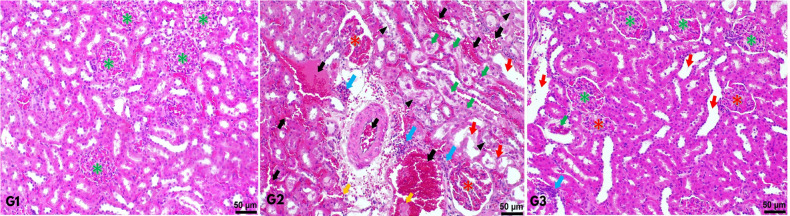
In Group 1, normal histological structures, including glomeruli and surrounding tubular structures, were observed without any tissue damage (G1). In Group 2, kidney tissues exhibited severe damage characterized by deformed glomerular and tubular structures, congestion and extensive hemorrhage, ischemia, parenchymal and stromal tissue infarction areas, intense inflammation, loss of brush borders in most tubules, apoptotic cells, and tubular cell debris (G2). In Group 3, moderate inflammation, tubular dilatation, and minimal glomerular deformities were observed. Green star: Represents normal glomerular structure. Red star: Represents deformed glomeruli. Red arrow: Represents tubular dilatation. Blue arrow: Represents inflammation. Black arrow: Represents hemorrhage and congestion. Green arrow: Represents apoptotic cells and tubular epithelial debris. Arrowhead: Represents ischemic and necrotic areas (Hematoxylin-eosin, Scale bar: 50 μm).

**Figure 4:**
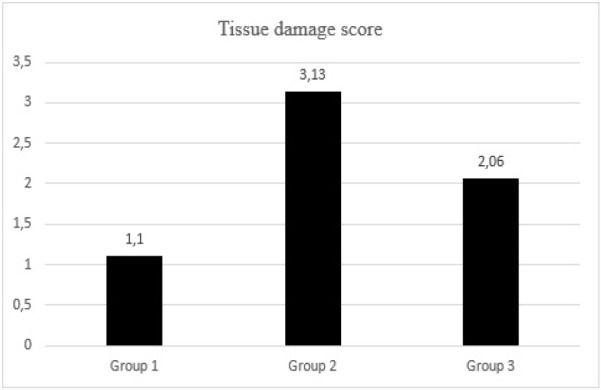
Comparative graphic representation of tissue damage score.

In Group 2, significant histopathological tissue damage was observed, including epithelial flattening, tubular dilation and irregularities, nuclear detachment/extrusion, loss of the brush border, ischemic and necrotic regions, glomerular deformities, localized tissue infarction areas, extensive hemorrhage, and congestion (Figure 3). The tissue damage score in Group 2 was recorded as 3.13±0.4, which was significantly elevated compared to the other groups (*P* < 0.001). Conversely, Group 3 exhibited notable improvement in renal tissue morphology under microscopic examination, with a recorded damage score of 2.06±0.26 (*P* < 0.001) (Table 3, Figure 4).

## Discussion

In this study, the effect of sugammadex on renal ischemia-reperfusion injury was investigated biochemically and histopathologically. Current biochemical results showed that sugammadex significantly suppressed the increase in MDA level and pro-inflammatory cytokine levels, including IL-1beta, IL-6, and TNF-alpha caused by the ischemia-reperfusion injury in renal tissue. Furthermore, it was revealed that sugammadex prevented the decrease in GSH-Px and SOD antioxidants caused by ischemia-reperfusion in renal tissue, and provided a significant improvement in renal function parameters such as NGAL and KIM-1. Sugammadex also exhibited notable recovery in renal tissue morphology under microscopic examination.

Acute kidney injury (AKI) is a medical condition defined by a rapid decline in renal function, occurring within hours to days. This impairment leads to the kidney’s an inability to efficiently eliminate nitrogenous waste products and regulate electrolyte and fluid balance. Each year, AKI affects an estimated 13.3 million individuals worldwide, with approximately 1.7 million associated fatalities annually.^19^ There are many factors involved in the etiology of acute renal failure, such as urinary stone diseases, infective pathologies, and the use of toxic pharmacological agents; however, renal ischemia–reperfusion injury is shown to be one of the most critical causes.^1^

The phenomenon of ischemia-reperfusion was first observed in the heart by Murry in 1986.^20^ However, we still have limited knowledge about the physiopathology of ischemia-reperfusion injury. During the ischemic period, anaerobic metabolism prevails since oxygen levels are quite low in the tissues. In this condition, tissues are not provided with high energy levels as in aerobic metabolism. Acidosis occurs in the cell due to lactate accumulation within the cellular environment.

The Na⁺/K⁺-ATPase pump is inhibited with decreased adenosine triphosphate (ATP) and pH levels. As a result, Na⁺ and Ca^2+^ ion concentrations increase inside the cell. Cellular edema develops because of this change in Na⁺ ions. Elevated Ca^2+^ activates protease and phosphatase, causing a cytotoxic effect on the cell. Furthermore, the lysosome membrane is destabilized, leading to the release of enzymes responsible for hydrolysis, which has detrimental effects on the cell in this acidic environment with low levels of energy. With the continuation of the ischemic process, an increase in the level of proinflammatory cytokines and a decrease in antioxidant enzyme levels are observed. This causes the cell to be highly vulnerable to tissue damage during the reperfusion period.

Low-energy-capacity adenosine monophosphates, and adenosine molecules, which are ATP products, are present in large amounts in the environment during the ischemic period. The breakdown of these molecules leads to elevated levels of hypoxanthine and inosine. When blood flow is reestablished in the tissues, oxygen, which is readily available in the environment, interacts with various molecules, particularly hypoxanthine that accumulates under ischemic conditions, and becomes unstable due to its reaction with oxygen-derived oxidants. This interaction triggers the formation of reactive oxygen species (ROS).^6,21^ A rapid surge in ROS levels plays a crucial role in renal ischemia-reperfusion injury. ROS levels are critical to maintaining cell life, affecting components such as membrane lipids, macromolecules, carbohydrates, and cell genomes. They contribute to cell apoptosis and necrosis via reversible or irreversible reactions with these biomolecules. Nevertheless, their ROS stimulate the production of vasoconstrictor mediators, such as endothelin-1 (ET-1). Accordingly, glomerular filtration levels are negatively affected.^4^

Extensive research has been conducted on renal ischemia-reperfusion injury over the past century. Cámara-Lemarroy et al.^5^ reported that reperfusion injury led to elevated levels of blood urea nitrogen (BUN), intercellular adhesion molecule-1, TNF-alpha, and ET-1, along with pronounced tubular damage. Similarly, Onem et al.^6^ found that renal ischemia-reperfusion injury adversely impacted kidney function, resulting in increased serum urea and creatinine levels. In our study, an increase was observed in NGAL and KIM-1 levels, which are important markers of renal function due to renal ischemia-reperfusion injury. Conversely, our histopathologic examinations showed that edema, inflammatory cell migration, tubular dilatation, and vascularization scores were all adversely affected. Choi et al.^22^ indicated that renal ischemia-reperfusion injury was associated with elevated levels of superoxide, nitric oxide, and peroxynitrite, ultimately contributing to severe renal tissue damage. Similarly, Salahshoor et al.^2^ observed an increase in lipid peroxidation following renal ischemia-reperfusion injury. Our analysis demonstrated that ischemia-reperfusion injury induced a prominent increase in MDA levels in experimental animals, alongside a significant decline in SOD and GSH-Px activity.

An acute inflammatory response occurs in ischemia-reperfusion injury. In the renal tissue exposed to oxidative damage, complex changes in the immune system, such as leukocyte activation, invasion, adhesion, and aggregation, are observed. This inflammatory reaction chain is a key contributor to the pathogenesis of renal ischemia-reperfusion injury.^4,20^

Oruc et al.^23^ demonstrated in their experimental study that ischemia-reperfusion injury led to a significant rise in neutrophil accumulation, myeloperoxidase enzyme activity, and lipid peroxidation levels. These biochemical and cellular alterations contributed to secondary damage, particularly affecting the glomeruli and renal tubules. Xu et al.^24^ reported that ischemia-reperfusion injury in the kidney was associated with elevated levels of BUN, creatinine, MDA, TNF-alpha, IL-1beta and IL-6, while simultaneously leading to a reduction in SOD and catalase activity. Consistent with previous findings, our study revealed a rise in proinflammatory cytokines, including TNF-alpha, IL-1beta and IL-6, after renal ischemia-reperfusion injury. Additionally, extensive inflammatory activity was observed within the renal tissue.

Sugammadex is a drug commonly used in anaesthesia practice that chemically binds aminosteroid neuromuscular blocking agents, providing rapid elimination and reversal of neuromuscular blockade. Residual neuromuscular blockade occurs after surgery, with an estimated 30 to 60% incidence in the recovery room.^25^ The low-level neuromuscular blockade (lower than what can be observed with the naked eye) has been linked to supralaryngeal muscle weakness that predisposes to impaired swallowing, upper airway obstruction, hypoxia, and an increased risk for aspiration. Sugammadex is a significant pharmacological agent because it increases the speed of reversal of neuromuscular blockade and thus minimizes all these risks.^26^ Sugammadex is not metabolized by the liver; therefore, liver function should not affect the drug’s pharmacokinetic profile. By contrast, sugammadex is primarily excreted in an unchanged form via urine within 24 hours at usual clinical doses, which makes it effective for rapid recovery from neuromuscular blockade.^27^ Administration of sugammadex in doses ranging from 2 mg kg^-1^ to 16 mg kg^-1^ demonstrates a linear and dose-dependent pharmacokinetic relationship, with an elimination half-life of 100 to 150 minutes and nearly 100% renal clearance.^28, 29^ The geometric mean time (95% confidence interval) from sugammadex administration to recovery of train-of-four (TOF) ratio to 0.9 increased with age: from 2.3 (2-2.6) minutes for younger adults to 2.6 (2.3-2.9) minutes for elderly adults and 3.6 (3.1-4.1) minutes for older adults.^27^ The most important indication for sugammadex in anaesthesia practice is its ability to provide fast and effective reversal in cases of difficult airway ventilation.^9^ A systematic review has indicated that the administration of sugammadex is associated with a decreased occurrence of pulmonary complications following surgery.^30^ Significant adverse effects with sugammadex have included bradycardia and anaphylaxis, with an incidence of anaphylaxis of 0.039%.^31^

Recent studies suggest that sugammadex serves as a potent pharmacological agent in mitigating oxidative stress in experimental ischemia-reperfusion models applied to different tissue types.^11^ However, the molecular mechanism of this situation has not been clearly explained.^32^ Sugammadex increases the production of apoptosis-inducing factor, caspase 3 protein, and monoclonal cytochrome c protein in cell cultures. They proposed that this role of sugammadex may be related to changing cholesterol homeostasis alongside oxidative stress and apoptosis.^32, 33^ On the other hand, it has been documented in many experimental studies that sugammadex prevents tissue damage against ischemia reperfusion injury by maintaining antioxidant levels, preventing polymorphonuclear leucocyte infiltration, reducing myeloperoxidase production, and decreasing inflammatory cytokine levels.^10, 11, 32^

Koç et al.^32^ observed, in a gastric ischemia-reperfusion model, that sugammadex suppressed levels of pro-inflammatory cytokines, such as TNF-alpha and IL-1beta, reduced myeloperoxidase activity-a lysosomal enzyme secreted by leukocytes in response to oxidative stress-and prevented the decline in total GSH levels as a result of ischemia-reperfusion injury. In their experimental study, creating an ovarian ischemia-reperfusion model, Kadioğlu et al.^11^ documented that sugammadex inhibited polymorphonuclear leukocyte infiltration in ovarian tissue, increased levels of antioxidant enzymes, such as GSH, SOD, and catalase, and prevented the increase in MDA levels. Consistent with our findings, MDA levels declined, while GSH and SOD activities increased in rats administered sugammadex. According to Alagöz et al.,^10^ sugammadex played a protective role against oxidative stress in a rat model of lower limb ischemia-reperfusion by preventing inflammatory cell formation. Yesiltas et al.^34^ also noted that sugammadex mitigated allergic inflammatory responses induced by rocuronium in rat lung tissue. Histopathological analysis in our study demonstrated a marked decrease in inflammation scores among experimental animals receiving sugammadex treatment. Additionally, a marked improvement pattern was observed in other tissue damage parameters, such as tubular dilatation and glomerular deformities, following sugammadex administration.

The optimal dose of sugammadex for the protective effect in ischemia-reperfusion injury is controversial. Although the protective efficacy of sugammadex against ischemia reperfusion injury has been analyzed in different dose ranges in the literature, we observed that the 4 mg kg^-1^ dose (similar to the usual application dose) was preferred frequently. In their experimental studies analyzing unilateral lower extremity ischemia reperfusion injury in rats, Alagöz et al.,^10^ observed that a dose of 4 mg kg^-1^ sugammadex protected against oxidative damage more effectively than a dose of 16 mg kg^-1^. Similarly, Koç et al.^32^ documented that 4 mg kg^-1^ sugammadex had effective anti-inflammatory and antioxidant effects in gastric ischemia-reperfusion injury. Kadioglu et al.^11^ reported that 4 mg kg^-1^ and 8 mg kg^-1^ doses of sugammadex minimized oxidative and inflammatory damage in ovarian ischemia reperfusion injury; however, 8 mg kg^-1^ was more effective. In the study of Tercan et al.,^35^ it was shown histopathologically that a high dose of 100 mg kg^-1^ sugammadex had a renoprotective effect on renal ischemia reperfusion injury. In our study, a 4 mg kg^-1^ sugamedex dose was preferred because its effective protection against oxidative damage at low doses was documented in previous experimental studies.^10, 31^

In line with the findings of our experimental study, numerous reports in the literature have highlighted the neuroprotective properties of sugammadex across different ischemia-reperfusion models.^33, 36^ In the cerebral ischemia-reperfusion model of Ciftci et al.,^36^ it is documented that the caspase-3 apopptotic cell numbers that increase due to ischemia-reperfusion damage were significantly reduced by sugammadex application. they reported that sugammadex suppressed the increased MDA and myeloperoxidase levels due to ischemia reperfusion injury. However, no significant difference was found between the groups in terms of SOD levels. In our study, apoptotic indexes are not analyzed. However, in our study, unlike the study by Ciftci et al.,^36^ a significant improvement in antioxidant levels was observed after sugammadex administration. In a similar study, Ozbilgin et al.^33^, found that the hippocampus *terminal deoxynucleotidyl transferase dUTP nick end labeling*-TUNEL and caspase results in the sugamadex treatment groups were significantly lower than those of the ischemia-reperfusion injury group. However, antioxidant enzyme activity and MDA levels in tissues were not evaluated in their study. In our study, sugammadex provided a significant improvement in kidney tissue morphology in microscopic examination.

Nevertheless, only a single study has specifically investigated the impact of sugammadex on renal ischemia-reperfusion injury. Tercan et al.^35^ conducted an experimental study demonstrating the renoprotective properties of sugammadex through histopathological evaluation. However, this study did not analyze antioxidant enzyme activities or assess blood parameters indicative of renal functions.

In our study, for the first time in the literature, sugammadex demonstrated antioxidant enzyme activity in blood and tissue biochemical analyses within a renal ischemia-reperfusion model, significantly improving renal functions while suppressing inflammation. Additionally, histopathological evaluations revealed a marked improvement in tissue condition.

### Study Limitations

While our study demonstrates the beneficial effects of sugammadex, a key limitation is the insufficient detail regarding its molecular mechanism and dose-response curve. Additionally, our study is constrained by its primary focus on the early-phase effects of sugammadex treatment. Moreover, the effects of sugammadex on renal tissue could not be demonstrated in healthy rats. For this reason, the intrinsic effects of sugammadex on renal function independent of ischemia-reperfusion injury have not been determined.

## Conclusion

Our biochemical and histopathological findings indicate that sugammadex exhibits notable protective effects against renal ischemia-reperfusion injury. We believe that with further comprehensive experimental and randomized large-scale clinical studies, the beneficial use of sugammadex will be observed in cases where renal ischemia-reperfusion injuries can be predicted, such as in kidney surgery and cardiopulmonary bypass. On the other hand, only the short-term effects of sugammadex were analyzed in our study. In this context, we believe that the investigation of the potential long-term effects of sugammadex in the chronic phase of renal recovery will make important contributions to the literature.

## Ethics

**Ethics Committee Approval:** The experiment adhered to the National Institutes of Health guidelines for animal research and received approval from the Tokat Gaziosmanpaşa University Rectorate, Animal Experiments Local Ethics Committee (approval no.: 2024 HADYEK-13, date: 15.08.2024).

**Informed Consent:** Animal study.

## Figures and Tables

**Table 1. Effect of Sugammadex on Pro-inflammatory Cytokine Levels:** 

-	**Groups**	**N**	**Mean ± SD**	***P* values**	**η² (Partial Eta Sq.)**	**Post-hoc *P* values**
**TNF-a (ng/L)**	1	7	565.33±152.17	0.002*	**0.504**	1-2:0.002*
	2	7	806.97±94.1			**1-3:0.480**
	3	7	634.04±60.2			2-3:0.021*
**IL-1beta (pg/mL)**	1	7	683.64±111.51	0.006*	**0.473**	1-2:0.009*
	2	7	858.74±99.61			**1-3:0.964**
	3	7	697.09±78.73			2-3:0.016*
**IL-6 (ng/L)**	1	7	11.34±1.60	<0.001*	**0.736**	1-2:<0.001*
	2	7	20.68±3.65			**1-3:0.059**
	3	7	14.65±1.70			2-3:0.006*

*: The *P* value is significant at the 0.05 level.

SD, standard deviation; TNF-alpha, tumor necrosis factor-alpha; IL, interleukin.

**Table 2. Effect of Sugammadex on Renal Function and Oxidative Stress Levels:** 

-	**Groups**	**N**	**Mean ± SD**	***P* values**	**η² (Partial Eta Sq.)**	**Post-hoc *P* values**
**SOD (U g protein)**	**1**	7	48.04±13.62	0.003*	**0.483**	1-2:0.003*
	**2**	7	28.54±5.17			**1-3:0.767**
	**3**	7	44.5±7.55			2-3:0.015*
**GSH-Px (U g protein)**	**1**	7	7.27±0.65	<0.001*	**0.743**	1-2:<0.001*
	**2**	7	4.48±0.97			**1-3:0.094**
	**3**	7	6.39±0.54			2-3:<0.001*
**MDA (nmol g wet tissue)**	**1**	7	5.83±0.72	<0.001*	**0.673**	1-2:<0.001*
	**2**	7	9.56±1.81			**1-3:0.091**
	**3**	7	7.22±0.48			2-3:0.004*
**KIM-1 (ng protein)**	**1**	7	6.27±2.19	0.015*	**0.373**	1-2:0.024*
	**2**	7	8.81±1.1			**1-3:0.985**
	**3**	7	6.41±1.4			2-3:0.034*
**NGAL (ng protein)**	**1**	7	24.26±6.07	<0.001*	**0.685**	1-2:<0.001*
	**2**	7	44.91±8.86			**1-3:0.472**
	**3**	7	28.42±3.55			2-3:<0.001*

*: The *P* value is significant at the 0.05 level.

SD, standard deviation; SOD, superoxide dismutase; GSH-Px, glutathione peroxidase; MDA, malondialdehyde; KIM-1, kidney injury molecule-1; NGAL,

neutrophil gelatinase-associated lipocalin.

**Table 3. Effect of Sugammadex on Kidney Injury Histopathologically:** 

-	**Groups**	**N**	**Mean ± SD**	***P* values**	**η² (Partial Eta Sq.)**	**Post-hoc P values**
**Tissue damage score**	**1**	7	1.1±0.1	<0.001*	**0.912**	1-2:<0.001*
	**2**	7	3.13±0.4			1-3:<0.001*
	**3**	7	2.06±0.26			2-3:<0.001*

*: The *P* value is significant at the 0.05 level.

Interpretation of partial eta square value:

0.01<η²<0.06: Small effect size. The independent variable has a weak effect on the dependent variable.

0.06<η²<0.14: Medium effect size. The independent variable has a moderate effect on the variable variable.

η²>0.14: Large effect size. The independent variable has a strong effect on the variable variable.

SD, standard deviation.
